# 2,2,6,6-Tetra­methyl­piperidinium triisopropoxysilanethiol­ate

**DOI:** 10.1107/S1600536809042962

**Published:** 2009-10-23

**Authors:** Katarzyna Baranowska, Paweł Roman, Justyna Socha

**Affiliations:** a Department of Inorganic Chemistry, Faculty of Chemistry, Gdańsk University of Technology, 11/12 G. Narutowicz St., 80233 - PL Gdańsk, Poland

## Abstract

The crystal of the title compound, C_9_H_20_N^+^·C_9_H_21_O_3_SSi^−^, is built of aggregates, each made up of two 2,2,6,6-tetra­methyl­piperidinium cations and two triisopropoxysilanethiol­ate anions. The aggregates are linked by four N—H⋯S bonds and correspond to an *R* ^2^_4_(8) graph-set motif.

## Related literature

For the structures of similar compounds and comparison of bond distances, see Baranowska, Chojnacki, Gosiewska & Wojnowski (2006 ▶); Baranowska, Chojnacki, Konitz *et al.* (2006 ▶); Baranowska & Piwowarska (2008 ▶); Becker *et al.* (2004 ▶). For the graph-set description of hydrogen-bonding patterns, see Bernstein *et al.* (1995 ▶); Etter (1990 ▶).
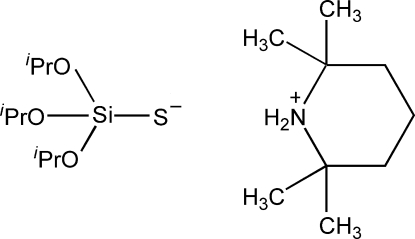


## Experimental

### 

#### Crystal data

C_9_H_20_N^+^·C_9_H_21_O_3_SSi^−^
*M* *_r_* = 379.67
Triclinic, 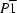

*a* = 9.2433 (6) Å
*b* = 11.5545 (8) Å
*c* = 11.7593 (7) Å
α = 85.955 (5)°
β = 77.190 (6)°
γ = 67.620 (6)°
*V* = 1132.28 (13) Å^3^
*Z* = 2
Mo *K*α radiation
μ = 0.21 mm^−1^
*T* = 120 K
0.42 × 0.39 × 0.33 mm

#### Data collection

Oxford Diffraction KM4/Xcalibur diffractometer with Sapphire2 detector
Absorption correction: analytical (*CrysAlis RED*; Oxford Diffraction, 2006 ▶) *T*_min_ = 0.908, *T*_max_ = 0.934
6937 measured reflections
4209 independent reflections
3561 reflections with *I* > 2σ(*I*)
*R*_int_ = 0.015

#### Refinement

*R*[*F* ^2^ > 2σ(*F* ^2^)] = 0.049
*wR*(*F* ^2^) = 0.122
*S* = 1.10
4209 reflections
235 parameters
1 restraint
H atoms treated by a mixture of independent and constrained refinement
Δρ_max_ = 0.45 e Å^−3^
Δρ_min_ = −0.43 e Å^−3^

### 

Data collection: *CrysAlis CCD* (Oxford Diffraction, 2006 ▶); cell refinement: *CrysAlis RED* (Oxford Diffraction, 2006 ▶); data reduction: *CrysAlis RED*; program(s) used to solve structure: *SHELXS97* (Sheldrick, 2008 ▶); program(s) used to refine structure: *SHELXL97* (Sheldrick, 2008 ▶); molecular graphics: *ORTEP-3 for Windows* (Farrugia, 1997 ▶); software used to prepare material for publication: *WinGX* publication routines (Farrugia, 1999 ▶).

## Supplementary Material

Crystal structure: contains datablocks I, global. DOI: 10.1107/S1600536809042962/ya2106sup1.cif

Structure factors: contains datablocks I. DOI: 10.1107/S1600536809042962/ya2106Isup2.hkl

Additional supplementary materials:  crystallographic information; 3D view; checkCIF report

## Figures and Tables

**Table 1:** Hydrogen-bond geometry (Å, °)

*D*—H⋯*A*	*D*—H	H⋯*A*	*D*⋯*A*	*D*—H⋯*A*
N1—H1*A*⋯S1	0.966 (17)	2.352 (17)	3.3166 (14)	177 (2)
N1—H1*B*⋯S1^i^	0.927 (17)	2.338 (17)	3.2354 (14)	163.0 (17)

Symmetry code: (i) 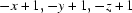.

**Fig. 1.**
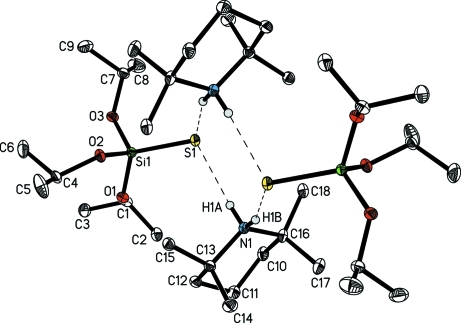
Dimeric aggregates [C9H20N+C9H21O3SiS-]2 in the structure of the title compound. The unlabeled atoms are derived from the reference atoms by the (1 - x, 1 - y, 1 - z) symmetry transformation. Displacement ellipsoids are drawn at the 50% probability level. C-bound H atoms have been omitted for clarity.

## supplementary crystallographic information

### Comment

Hydrogen bonding is arguably the most prominent interaction in selfassembly of molecules in crystals and plays an important role in determining structure of chemical and biological systems. Hydrogen bonds of the ^(+)^N···H–S^(–)^type have gained relatively little attention, as these bonds are mostly weak and quite seldom lead to the proton transfer. One notable exception are silanethiolates, where ionization of the SH group is facilitated by the neighbouring silicon atom. The salts of these anions with primary amines as counter-ions often feature tetrameric aggregates with a cubane-like hydrogen bonded core (Becker *et al.*, 2004). Secondary amines give derivatives with discrete dimeric units in the solid state (Baranowska, Chojnacki, Konitz *et al. *2006).

We present here the crystal structure of the title compound, which was obtained by the reaction of tri-*iso*-propoxysilanethiol with 2,2,6,6-tetramethylpiperidine.

The crystal structure is built of aggregates, made up of two tri-*iso*-propoxysilanethiolate anions and two piperidinium cations (Fig. 1). The aggregates contain eight-membered ring built due to the formation of four charge-assisted ^(+)^*N*–*H*···*S*^(–)^hydrogen bonds (graph theory motif *R*^2^_4_(8) according to Etter, 1990; Bernstein *et al.*, 1995). The N···S distances (Table 1) lie in the range comparable with the values observed in aromatic thiolates (Baranowska & Piwowarska, 2008) or silanethiolates (Baranowska, Chojnacki, Gosiewska, Wojnowski, 2006).

### Experimental

Tri-*iso*-propoxysilanethiol (2 mmol) was dissolved in 8 ml of propanol-2 and 2,2,6,6-tetramethylpiperidine (0,338 ml, 2 mmol) was added. The solvent was then added gradually until a white deposit formed was completely dissolved. The solution was left to stand at 4 °C for a few days for crystallization. The obtained colourless crystals were suitable for X-ray diffraction analysis. The product is hygroscopic and slowly oxidizes in the air, therefore all operations were carried out using a vacuum-nitrogen line and Schlenk techniques.

### Refinement

Hydrogen atoms were placed in geometrically calculated positions (C—H 0.98 Å for methyl, 0.99 Å for methylene and 1.00 Å for methine H atoms) and refined as riding on their parent atoms with *U*_iso_(H) = 1.2*U*_eq_(C) for methylene and methine and 1.5*U*_eq_(C) for methyl groups. Hydrogen atoms of ammonium group were found in the difference map and refined in isotropic approximation constrained to produce N—H bonds equal within 0.04 Å (SADI instruction of *SHELXL97*; Sheldrick, 2008)

### Crystal data

**Table:**

C_9_H_20_N^+^·C_9_H_21_O_3_SSi^−^	*Z* = 2
*M**_r_* = 379.67	*F*(000) = 420
Triclinic, *P*1	*D*_x_ = 1.114 Mg m^−^^3^
Hall symbol: -P 1	Mo *K*α radiation, λ = 0.71073 Å
*a* = 9.2433 (6) Å	Cell parameters from 5191 reflections
*b* = 11.5545 (8) Å	θ = 2.4–28.8°
*c* = 11.7593 (7) Å	µ = 0.21 mm^−^^1^
α = 85.955 (5)°	*T* = 120 K
β = 77.190 (6)°	Prism, colourless
γ = 67.620 (6)°	0.42 × 0.39 × 0.33 mm
*V* = 1132.28 (13) Å^3^	

### Data collection

**Table:**

Oxford Diffraction KM4/Xcalibur diffractometer with Sapphire2 detector	4209 independent reflections
graphite	3561 reflections with *I* > 2σ(*I*)
Detector resolution: 8.1883 pixels mm^-1^	*R*_int_ = 0.015
ω scans	θ_max_ = 25.5°, θ_min_ = 2.4°
Absorption correction: analytical (*CrysAlis RED*; Oxford Diffraction, 2006)	*h* = −10→11
*T*_min_ = 0.908, *T*_max_ = 0.934	*k* = −13→13
6937 measured reflections	*l* = −7→14

### Refinement

**Table:**

Refinement on *F*^2^	Primary atom site location: structure-invariant direct methods
Least-squares matrix: full	Secondary atom site location: difference Fourier map
*R*[*F*^2^ > 2σ(*F*^2^)] = 0.049	Hydrogen site location: inferred from neighbouring sites
*wR*(*F*^2^) = 0.122	H atoms treated by a mixture of independent and constrained refinement
*S* = 1.10	*w* = 1/[σ^2^(*F*_o_^2^) + (0.0855*P*)^2^] where *P* = (*F*_o_^2^ + 2*F*_c_^2^)/3
4209 reflections	(Δ/σ)_max_ < 0.001
235 parameters	Δρ_max_ = 0.45 e Å^−^^3^
1 restraint	Δρ_min_ = −0.43 e Å^−^^3^

### Special details

**Table:**

Geometry. All s.u.'s (except the s.u. in the dihedral angle between two l.s. planes) are estimated using the full covariance matrix. The cell s.u.'s are taken into account individually in the estimation of s.u.'s in distances, angles and torsion angles; correlations between s.u.'s in cell parameters are only used when they are defined by crystal symmetry. An approximate (isotropic) treatment of cell s.u.'s is used for estimating s.u.'s involving l.s. planes.
Refinement. Refinement of *F*^2^ against ALL reflections. The weighted *R*-factor *wR* and goodness of fit *S* are based on *F*^2^, conventional *R*-factors *R* are based on *F*, with *F* set to zero for negative *F*^2^. The threshold expression of *F*^2^ > 2σ(*F*^2^) is used only for calculating *R*-factors(gt) *etc*. and is not relevant to the choice of reflections for refinement. *R*-factors based on *F*^2^ are statistically about twice as large as those based on *F*, and *R*- factors based on ALL data will be even larger.

### Fractional atomic coordinates and isotropic or equivalent isotropic displacement parameters (Å^2^)

**Table:**

	*x*	*y*	*z*	*U*_iso_*/*U*_eq_	
C1	0.7770 (2)	0.42248 (16)	−0.00766 (13)	0.0205 (4)	
H1	0.7182	0.3662	−0.0104	0.025*	
C2	0.6713 (2)	0.55565 (17)	−0.02787 (15)	0.0272 (4)	
H2A	0.5712	0.5799	0.031	0.041*	
H2B	0.6476	0.5616	−0.1058	0.041*	
H2C	0.7266	0.6117	−0.022	0.041*	
C3	0.9338 (2)	0.37720 (18)	−0.09727 (15)	0.0302 (4)	
H3A	0.9935	0.4305	−0.0932	0.045*	
H3B	0.9115	0.3814	−0.1755	0.045*	
H3C	0.9975	0.2905	−0.0809	0.045*	
C4	1.0748 (2)	0.28839 (16)	0.23790 (15)	0.0236 (4)	
H4	1.0715	0.3583	0.182	0.028*	
C5	1.1457 (3)	0.3044 (3)	0.33692 (19)	0.0506 (6)	
H5A	1.0774	0.3838	0.3781	0.076*	
H5B	1.253	0.3049	0.3057	0.076*	
H5C	1.1527	0.235	0.3912	0.076*	
C6	1.1706 (3)	0.1663 (2)	0.1731 (2)	0.0481 (6)	
H6A	1.1779	0.0969	0.2274	0.072*	
H6B	1.2783	0.1635	0.1377	0.072*	
H6C	1.1179	0.1587	0.1119	0.072*	
C7	0.7501 (2)	0.09780 (16)	0.18044 (15)	0.0246 (4)	
H7	0.6694	0.1273	0.255	0.03*	
C8	0.6687 (3)	0.0770 (2)	0.0905 (2)	0.0460 (6)	
H8A	0.5784	0.1542	0.0818	0.069*	
H8B	0.6295	0.0096	0.1156	0.069*	
H8C	0.7452	0.0538	0.0156	0.069*	
C9	0.8863 (3)	−0.01829 (18)	0.2035 (2)	0.0416 (5)	
H9A	0.9654	−0.0482	0.1306	0.062*	
H9B	0.8453	−0.0834	0.2343	0.062*	
H9C	0.9369	0.0013	0.2605	0.062*	
C10	0.3378 (2)	0.77040 (17)	0.24261 (14)	0.0256 (4)	
H10A	0.3714	0.6889	0.2026	0.031*	
H10B	0.2393	0.828	0.2184	0.031*	
C11	0.4694 (2)	0.82307 (17)	0.20331 (15)	0.0280 (4)	
H11A	0.4912	0.8302	0.1173	0.034*	
H11B	0.4333	0.9078	0.2376	0.034*	
C12	0.6221 (2)	0.73804 (16)	0.24148 (14)	0.0233 (4)	
H12A	0.705	0.7745	0.216	0.028*	
H12B	0.6617	0.6555	0.202	0.028*	
C13	0.5987 (2)	0.71909 (15)	0.37349 (14)	0.0197 (4)	
C14	0.5719 (2)	0.83674 (16)	0.44152 (16)	0.0290 (4)	
H14A	0.5284	0.8278	0.5242	0.044*	
H14B	0.496	0.9102	0.4105	0.044*	
H14C	0.6739	0.8473	0.4333	0.044*	
C15	0.7431 (2)	0.61143 (15)	0.40297 (15)	0.0234 (4)	
H15A	0.8393	0.6312	0.3766	0.035*	
H15B	0.7574	0.5346	0.3637	0.035*	
H15C	0.7255	0.5993	0.4875	0.035*	
C16	0.2995 (2)	0.75210 (16)	0.37445 (14)	0.0218 (4)	
C17	0.2107 (2)	0.87568 (17)	0.44395 (16)	0.0319 (4)	
H17A	0.2099	0.8596	0.527	0.048*	
H17B	0.1004	0.9126	0.4327	0.048*	
H17C	0.265	0.9337	0.4166	0.048*	
C18	0.1976 (2)	0.67189 (17)	0.40504 (15)	0.0255 (4)	
H18A	0.2555	0.5901	0.3656	0.038*	
H18B	0.0963	0.7139	0.3795	0.038*	
H18C	0.1757	0.6603	0.4896	0.038*	
O1	0.81444 (14)	0.41560 (10)	0.10576 (9)	0.0198 (3)	
O2	0.91262 (14)	0.29862 (11)	0.28713 (10)	0.0212 (3)	
O3	0.81113 (14)	0.19313 (10)	0.13885 (10)	0.0233 (3)	
Si1	0.77654 (5)	0.32207 (4)	0.20952 (4)	0.01653 (15)	
S1	0.55226 (5)	0.38899 (4)	0.31590 (3)	0.01896 (14)	
N1	0.45724 (16)	0.67745 (12)	0.41129 (12)	0.0182 (3)	
H1A	0.489 (3)	0.5932 (17)	0.3823 (19)	0.046 (6)*	
H1B	0.437 (2)	0.6748 (18)	0.4920 (15)	0.033 (5)*	

### Atomic displacement parameters (Å^2^)

**Table:**

	*U*^11^	*U*^22^	*U*^33^	*U*^12^	*U*^13^	*U*^23^
C1	0.0237 (9)	0.0253 (9)	0.0159 (8)	−0.0128 (8)	−0.0048 (7)	0.0011 (6)
C2	0.0229 (9)	0.0336 (10)	0.0226 (9)	−0.0078 (8)	−0.0055 (7)	0.0023 (7)
C3	0.0304 (11)	0.0329 (10)	0.0224 (9)	−0.0099 (9)	0.0024 (8)	−0.0050 (7)
C4	0.0152 (9)	0.0275 (9)	0.0286 (9)	−0.0100 (8)	−0.0036 (7)	0.0046 (7)
C5	0.0249 (11)	0.0914 (19)	0.0412 (13)	−0.0269 (13)	−0.0081 (9)	−0.0038 (12)
C6	0.0200 (10)	0.0409 (12)	0.0749 (17)	−0.0051 (10)	−0.0006 (11)	−0.0118 (11)
C7	0.0254 (10)	0.0226 (9)	0.0265 (9)	−0.0123 (8)	0.0003 (7)	−0.0025 (7)
C8	0.0505 (15)	0.0505 (13)	0.0541 (14)	−0.0334 (12)	−0.0207 (12)	0.0073 (11)
C9	0.0400 (13)	0.0258 (10)	0.0625 (14)	−0.0147 (10)	−0.0153 (11)	0.0079 (9)
C10	0.0215 (9)	0.0296 (9)	0.0209 (9)	−0.0043 (8)	−0.0050 (7)	0.0022 (7)
C11	0.0295 (10)	0.0269 (9)	0.0220 (9)	−0.0068 (8)	−0.0033 (8)	0.0055 (7)
C12	0.0238 (9)	0.0233 (9)	0.0223 (9)	−0.0107 (8)	−0.0007 (7)	0.0015 (7)
C13	0.0200 (9)	0.0202 (8)	0.0199 (8)	−0.0094 (7)	−0.0023 (7)	−0.0015 (6)
C14	0.0344 (11)	0.0262 (9)	0.0288 (10)	−0.0140 (9)	−0.0044 (8)	−0.0067 (7)
C15	0.0199 (9)	0.0249 (9)	0.0272 (9)	−0.0096 (8)	−0.0059 (7)	−0.0013 (7)
C16	0.0168 (8)	0.0223 (9)	0.0205 (8)	−0.0010 (7)	−0.0036 (7)	−0.0007 (7)
C17	0.0279 (10)	0.0267 (10)	0.0301 (10)	0.0005 (8)	−0.0022 (8)	−0.0041 (8)
C18	0.0171 (9)	0.0312 (10)	0.0252 (9)	−0.0061 (8)	−0.0038 (7)	−0.0003 (7)
O1	0.0239 (6)	0.0233 (6)	0.0161 (6)	−0.0124 (5)	−0.0057 (5)	0.0009 (5)
O2	0.0149 (6)	0.0279 (6)	0.0208 (6)	−0.0084 (5)	−0.0036 (5)	0.0028 (5)
O3	0.0258 (7)	0.0200 (6)	0.0230 (6)	−0.0114 (5)	0.0034 (5)	−0.0040 (5)
Si1	0.0155 (3)	0.0168 (2)	0.0167 (2)	−0.00606 (19)	−0.00172 (18)	−0.00110 (17)
S1	0.0151 (2)	0.0219 (2)	0.0187 (2)	−0.00605 (18)	−0.00163 (16)	−0.00349 (16)
N1	0.0169 (7)	0.0187 (7)	0.0178 (7)	−0.0053 (6)	−0.0033 (6)	−0.0006 (6)

### Geometric parameters (Å, °)

**Table:**

C1—O1	1.4399 (18)	C10—H10A	0.99
C1—C2	1.512 (2)	C10—H10B	0.99
C1—C3	1.519 (2)	C11—C12	1.523 (2)
C1—H1	1.00	C11—H11A	0.99
C2—H2A	0.98	C11—H11B	0.99
C2—H2B	0.98	C12—C13	1.531 (2)
C2—H2C	0.98	C12—H12A	0.99
C3—H3A	0.98	C12—H12B	0.99
C3—H3B	0.98	C13—C15	1.526 (2)
C3—H3C	0.98	C13—N1	1.527 (2)
C4—O2	1.444 (2)	C13—C14	1.536 (2)
C4—C6	1.502 (3)	C14—H14A	0.98
C4—C5	1.510 (2)	C14—H14B	0.98
C4—H4	1.00	C14—H14C	0.98
C5—H5A	0.98	C15—H15A	0.98
C5—H5B	0.98	C15—H15B	0.98
C5—H5C	0.98	C15—H15C	0.98
C6—H6A	0.98	C16—N1	1.525 (2)
C6—H6B	0.98	C16—C18	1.531 (2)
C6—H6C	0.98	C16—C17	1.535 (2)
C7—O3	1.430 (2)	C17—H17A	0.98
C7—C8	1.506 (3)	C17—H17B	0.98
C7—C9	1.507 (3)	C17—H17C	0.98
C7—H7	1.00	C18—H18A	0.98
C8—H8A	0.98	C18—H18B	0.98
C8—H8B	0.98	C18—H18C	0.98
C8—H8C	0.98	O1—Si1	1.6408 (11)
C9—H9A	0.98	O2—Si1	1.6441 (11)
C9—H9B	0.98	O3—Si1	1.6452 (11)
C9—H9C	0.98	Si1—S1	2.0558 (6)
C10—C11	1.530 (3)	N1—H1A	0.966 (17)
C10—C16	1.530 (2)	N1—H1B	0.927 (17)
			
O1—C1—C2	108.93 (13)	C10—C11—H11A	109.6
O1—C1—C3	107.87 (14)	C12—C11—H11B	109.6
C2—C1—C3	112.55 (14)	C10—C11—H11B	109.6
O1—C1—H1	109.1	H11A—C11—H11B	108.1
C2—C1—H1	109.1	C11—C12—C13	113.24 (15)
C3—C1—H1	109.1	C11—C12—H12A	108.9
C1—C2—H2A	109.5	C13—C12—H12A	108.9
C1—C2—H2B	109.5	C11—C12—H12B	108.9
H2A—C2—H2B	109.5	C13—C12—H12B	108.9
C1—C2—H2C	109.5	H12A—C12—H12B	107.7
H2A—C2—H2C	109.5	C15—C13—N1	105.89 (13)
H2B—C2—H2C	109.5	C15—C13—C12	110.41 (14)
C1—C3—H3A	109.5	N1—C13—C12	107.30 (12)
C1—C3—H3B	109.5	C15—C13—C14	108.82 (13)
H3A—C3—H3B	109.5	N1—C13—C14	111.15 (14)
C1—C3—H3C	109.5	C12—C13—C14	113.03 (14)
H3A—C3—H3C	109.5	C13—C14—H14A	109.5
H3B—C3—H3C	109.5	C13—C14—H14B	109.5
O2—C4—C6	111.26 (14)	H14A—C14—H14B	109.5
O2—C4—C5	107.29 (15)	C13—C14—H14C	109.5
C6—C4—C5	112.44 (17)	H14A—C14—H14C	109.5
O2—C4—H4	108.6	H14B—C14—H14C	109.5
C6—C4—H4	108.6	C13—C15—H15A	109.5
C5—C4—H4	108.6	C13—C15—H15B	109.5
C4—C5—H5A	109.5	H15A—C15—H15B	109.5
C4—C5—H5B	109.5	C13—C15—H15C	109.5
H5A—C5—H5B	109.5	H15A—C15—H15C	109.5
C4—C5—H5C	109.5	H15B—C15—H15C	109.5
H5A—C5—H5C	109.5	N1—C16—C10	107.55 (13)
H5B—C5—H5C	109.5	N1—C16—C18	106.11 (13)
C4—C6—H6A	109.5	C10—C16—C18	110.72 (14)
C4—C6—H6B	109.5	N1—C16—C17	110.86 (13)
H6A—C6—H6B	109.5	C10—C16—C17	113.25 (14)
C4—C6—H6C	109.5	C18—C16—C17	108.14 (15)
H6A—C6—H6C	109.5	C16—C17—H17A	109.5
H6B—C6—H6C	109.5	C16—C17—H17B	109.5
O3—C7—C8	107.94 (15)	H17A—C17—H17B	109.5
O3—C7—C9	108.78 (15)	C16—C17—H17C	109.5
C8—C7—C9	113.02 (17)	H17A—C17—H17C	109.5
O3—C7—H7	109	H17B—C17—H17C	109.5
C8—C7—H7	109	C16—C18—H18A	109.5
C9—C7—H7	109	C16—C18—H18B	109.5
C7—C8—H8A	109.5	H18A—C18—H18B	109.5
C7—C8—H8B	109.5	C16—C18—H18C	109.5
H8A—C8—H8B	109.5	H18A—C18—H18C	109.5
C7—C8—H8C	109.5	H18B—C18—H18C	109.5
H8A—C8—H8C	109.5	C1—O1—Si1	124.60 (10)
H8B—C8—H8C	109.5	C4—O2—Si1	123.87 (10)
C7—C9—H9A	109.5	C7—O3—Si1	126.27 (11)
C7—C9—H9B	109.5	O1—Si1—O2	104.18 (6)
H9A—C9—H9B	109.5	O1—Si1—O3	103.66 (6)
C7—C9—H9C	109.5	O2—Si1—O3	110.48 (6)
H9A—C9—H9C	109.5	O1—Si1—S1	116.03 (5)
H9B—C9—H9C	109.5	O2—Si1—S1	109.64 (5)
C11—C10—C16	113.34 (14)	O3—Si1—S1	112.43 (5)
C11—C10—H10A	108.9	C16—N1—C13	119.89 (13)
C16—C10—H10A	108.9	C16—N1—H1A	105.6 (13)
C11—C10—H10B	108.9	C13—N1—H1A	108.5 (14)
C16—C10—H10B	108.9	C16—N1—H1B	108.0 (13)
H10A—C10—H10B	107.7	C13—N1—H1B	106.7 (13)
C12—C11—C10	110.38 (14)	H1A—N1—H1B	107.6 (18)
C12—C11—H11A	109.6		
			
C16—C10—C11—C12	57.4 (2)	C1—O1—Si1—O3	37.28 (14)
C10—C11—C12—C13	−57.91 (19)	C1—O1—Si1—S1	−86.47 (13)
C11—C12—C13—C15	167.08 (14)	C4—O2—Si1—O1	−35.98 (13)
C11—C12—C13—N1	52.13 (18)	C4—O2—Si1—O3	74.77 (13)
C11—C12—C13—C14	−70.76 (19)	C4—O2—Si1—S1	−160.78 (11)
C11—C10—C16—N1	−51.19 (19)	C7—O3—Si1—O1	−158.72 (12)
C11—C10—C16—C18	−166.70 (14)	C7—O3—Si1—O2	90.20 (13)
C11—C10—C16—C17	71.65 (19)	C7—O3—Si1—S1	−32.64 (14)
C2—C1—O1—Si1	123.01 (13)	C10—C16—N1—C13	50.41 (18)
C3—C1—O1—Si1	−114.55 (14)	C18—C16—N1—C13	168.93 (13)
C6—C4—O2—Si1	−72.47 (18)	C17—C16—N1—C13	−73.89 (18)
C5—C4—O2—Si1	164.15 (14)	C15—C13—N1—C16	−168.72 (13)
C8—C7—O3—Si1	126.51 (15)	C12—C13—N1—C16	−50.79 (18)
C9—C7—O3—Si1	−110.51 (16)	C14—C13—N1—C16	73.25 (17)
C1—O1—Si1—O2	152.92 (12)		

### Hydrogen-bond geometry (Å, °)

**Table:**

*D*—H···*A*	*D*—H	H···*A*	*D*···*A*	*D*—H···*A*
N1—H1A···S1	0.97 (2)	2.35 (2)	3.3166 (14)	177 (2)
N1—H1B···S1^i^	0.93 (2)	2.34 (2)	3.2354 (14)	163 (2)

Symmetry codes: (i) −*x*+1, −*y*+1, −*z*+1.
